# Development of the Nature Impact Mental Health Intervention for People Experiencing Mild to Moderate Anxiety, Depression, and/or Stress—Co-Producing a Programme Theory and Logic Model

**DOI:** 10.3390/healthcare14131861

**Published:** 2026-06-25

**Authors:** Louise S. Madsen, Dorthe V. Poulsen, Knud Ryom, Lisa Gregersen Oestergaard, Thomas Maribo, Nanna Holt Jessen

**Affiliations:** 1 DEFACTUM, Central Denmark Region, 8000 Aarhus, Denmark; lmaden@rm.dk (L.S.M.); lisaoest@rm.dk (L.G.O.); thomas.maribo@rm.dk (T.M.); 2 Department of Geosciences and Natural Resource Management, University of Copenhagen, 1165 Copenhagen, Denmark; dvp@ign.ku.dk; 3 Department of Public Health, Applied Public Health, Aarhus University, 8000 Aarhus, Denmark; knudryom@ph.au.dk; 4 Research Unit for General Practice, Aarhus University, 8000 Aarhus, Denmark

**Keywords:** nature-based health interventions, programme theory, mental health, anxiety, depression, stress

## Abstract

**Highlights:**

**What are the main findings?**

- A structured co-production process successfully developed a transparent programme theory for a nature-based health intervention (NBHI), including clearly defined mechanisms of change.
- The resulting logic model links intervention activities to intended mental health outcomes and provides a coherent, evidence-informed framework for further testing and application.

**What are the implications of the main findings?**

- The model offers a practical and transferable foundation for implementing and evaluating NBHIs within healthcare systems.
- The study provides methodological guidance for improving transparency, reproducibility, and integration of co-produced interventions in mental health care.

**Abstract:**

**Background:** Nature-based health interventions (NBHIs) show promising potential for supporting people experiencing mild to moderate anxiety, depression, and stress. However, their underlying programme theories are rarely made explicit, limiting transparency, implementation, and transferability within healthcare contexts. The Nature Impact Mental Health Intervention is a context-adapted, nature-based programme designed to support mental health and well-being. This article aims to describe its development through a structured co-production process and presents its programme theory and logic model. **Methods:** The co-production-based development process followed a three-stage framework. Stage 1 established a scientific foundation through a systematic review, stakeholder analysis, dialogue meetings, and a Delphi study to synthesise evidence and identify knowledge gaps. Stage 2 involved a co-production workshop with practice partners and researchers to translate evidence and refine intervention components. Stage 3 consolidated outputs and site visits into an operational intervention catalogue for prototyping the resulting programme theory and logic model. **Results:** The co-production process yielded a coherent programme theory comprising clearly defined mechanisms of change and aligned intervention activities. These were iteratively refined through workshops and prototyping, resulting in a consolidated logic model that articulates hypothesised causal pathways linking activities to outcomes. The model also provides a practical framework for guiding subsequent feasibility testing, implementation, and evaluation across contexts. **Conclusions:** This study demonstrates a transparent development process for co-producing a programme theory and logic model for NBHIs. The resulting model provides a theoretically grounded and implementation-sensitive foundation for subsequent feasibility testing and contributes methodological guidance for integrating NBHIs within healthcare systems.

## 1. Introduction

Anxiety, depression, and stress represent an escalating public health challenge globally, contributing substantially to reduced quality of life, impaired functioning, and increased societal and economic burden [1,2]. In Denmark, anxiety and depression affect a considerable proportion of the population, corresponding to approximately 3200 and 9900 individuals per 100,000, respectively. These conditions are associated with increased mortality, contributing to excess deaths at a population level, and lead to substantial use of primary care services, including a high number of general practitioner contacts. They also impose a considerable economic burden, with annual costs in the range of several hundred million to over a billion euros in healthcare, and substantially higher losses related to reduced productivity [3,4].

Despite a growing availability of evidence-based psychological and pharmacological treatments, many people experiencing mild to moderate symptoms do not access or benefit sufficiently from conventional mental health services [5]. Furthermore, barriers include limited service capacity, stigma, long waiting times, and a mismatch between people’s needs and the predominantly clinical, symptom-focused nature of existing care pathways [6,7]. These challenges have intensified global interest in complementary and alternative approaches to mental health support [7].

Nature-based health interventions (NBHIs) have emerged as a promising and increasingly studied approach within interventions targeting mental health challenges such as anxiety, depression, and/or stress [8,9,10,11]. These interventions have a therapeutic purpose, supporting mental, emotional, and physical well-being through structured or facilitated engagement with natural environments [8]. They typically include activities such as guided nature exposure, horticultural therapy (therapeutic activities involving the cultivation and care of plants), and mindful nature-based activities (intentional awareness practices such as mindful walking and sensory focused exercises). Emerging interventions are typically group-based and evidence-informed outdoor group programmes, carried out in green or blue spaces [8]. The growing interest in NBHIs is supported by theoretical perspectives that explain how contact with nature may promote psychological well-being. Attention Restoration Theory suggests that the many intense and competing stimuli of everyday urban life can fatigue the brain, while natural environments offer different, less demanding sensory inputs that support mental restoration and reduce stress [12,13].

Together, the different theories provide conceptual explanations for how engagement with nature may support mental health through several interrelated processes, including emotional regulation, attentional restoration, physical activity, and opportunities for social interaction. Consistent with these theoretical perspectives, a growing body of empirical research indicates that NBHIs are associated with reductions in symptoms of anxiety, depression, and stress, as well as improvements in overall well-being [8]. However, the evidence base remains heterogeneous, with considerable variation in intervention types, target populations, settings, and outcome measures [8,9,14,15].

One key challenge in advancing the field concerns the complexity and context sensitivity of NBHIs [16]. These interventions are typically multi-component, involving interactions between environmental, social, psychological, and physiological processes, and their effects may depend on individual characteristics, local contexts, and implementation conditions [16,17]. Despite this complexity, NBHIs are often described in relatively pragmatic or superficial terms within healthcare and public health contexts, with limited articulation of the underlying assumptions about how and why they are expected to produce beneficial outcomes [8,18]. This lack of explicitly articulated programme theories limits both scientific understanding and practical implementation [16]. Without a clear account of the hypothesised mechanisms of change, it is difficult to assess for whom NBHIs are most appropriate, under which conditions they are likely to be effective, and how they may be adapted or scaled while maintaining their intended effects [17]. In this context, participatory approaches, such as co-production, have gained increasing attention as both a methodological and practical approach [19]. Through systematic collaboration with practitioners and other stakeholders, co-production aims to enable the early identification of context-specific barriers and facilitators, iterative testing and refinement of materials, and the development of sustainable, replicable collaborative processes. Although evidence of its consistent impact is still emerging, co-production is expected to reduce the risk of implementing interventions that are poorly adapted to real-world settings [19].

The Nature Impact Mental Health Intervention was developed in response to these gaps. It is a context-adapted NBHI designed to support mental health and well-being among people experiencing mild to moderate anxiety, depression, and/or stress [16]. The intervention seeks to integrate evidence from environmental psychology, mental health, and public health research with practical considerations related to healthcare delivery and local context. By explicitly addressing the role of nature engagement within a structured yet flexible programme, the intervention aims to offer a feasible and acceptable supplement to existing mental health support, particularly in early or preventive stages of healthcare.

Therefore, the aim of this study was to describe the development of the Nature Impact Mental Health Intervention through a structured co-production process and to present its programme theory and logic model.

## 2. Materials and Methods

### 2.1. Study Context

Nature Impact is a national research project in Denmark starting 1 November 2023 that seeks to develop and feasibility-test a context-adapted, co-produced NBHI for people experiencing mild to moderate anxiety, depression, and/or stress within a structured healthcare context: The Nature Impact Mental Health Intervention. The project is conducted in collaboration with three intervention practice partners from two municipalities, and one outpatient clinic at a Psychiatric Centre, integrating research and real-world implementation perspectives.

### 2.2. Study Design

The study design was inspired by the three-stage co-production framework as suggested by Hawkins et al. [19]. The three stages in the current study include Stage (1) scientific foundation, (2) co-production workshop, and (3) prototyping and context adaptation.

**Figure 1:**
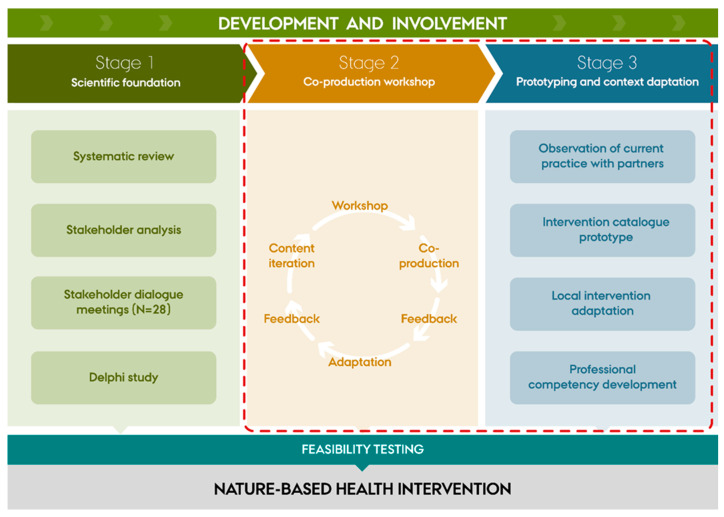
The framework and activities completed in the Nature Impact Mental Health Intervention development process using co-production. The red dotted line symbolises the focus of the current article. The figure is inspired by Figure 1, Madsen et al., 2026 [16].

Figure 1 illustrates the framework and activities completed in the Nature Impact Mental Health Intervention development process using co-production. The analytical emphasis of this article lies in the iterative development, refinement, and contextual adaptation of the programme theory and logic model across Stages 2 and 3. Across stages, the methods were designed to support the systematic integration of evidence from scientific literature with stakeholders, expert, practice-based, and experiential knowledge.

### 2.3. Summary of Key-Findings from Stage 1. Scientific Foundation

Stage 1 established the empirical and conceptual basis for the subsequent co-production process. Key insights described elsewhere are briefly revisited to situate the following stages [8,16]. It comprised four interrelated and complementary components (Figure 1) designed to synthesise existing evidence on NBHIs, incorporate stakeholder perspectives, and articulate expert assumptions regarding mechanisms, contextual conditions, and intended outcomes.

In the systematic review, forty-five different outcome measures were found grouped into four categories: symptom scales, overall mental health scales, nature and therapeutic scales, and other health outcomes [8]. The instruments were used across the 19 included articles, without clear guidance as to which measures were most appropriate for evaluating intervention effects in practice. The systematic review also identified preliminary evidence that NBHIs may improve symptoms of anxiety, depression and stress, as well as overall mental health. However, the evidence base was characterised by substantial heterogeneity and generally low methodological quality, highlighting the need for stronger theoretical frameworks and more robust evaluation approaches. These findings were synthesised into preliminary assumptions and focal questions that structured the subsequent stakeholder dialogues and Delphi process [16].

A stakeholder analysis informed the identification of key actors and the recruitment strategy through team brainstorming, expert consultations, and participatory mapping using the STICK-E software © Deakin University [20]. Stakeholders were assessed according to their level of interest and influence, informing the composition of stakeholder dialogue meetings and workshops. This approach supported the visualisation of system relationships and facilitated a shared understanding of the context in which the intervention operates. The key stakeholders in intervention development were the delivery teams of the three intervention practice partners, who were involved throughout Stage 1, 2, and 3. Other stakeholders were involved in Stage 1 and included people with lived experience, general practitioners, representatives from the Danish Nature Agency, NGOs, private nature–health stakeholders and researchers with expertise related to the mental health target group. Key actors were subsequently selected through purposive sampling [21]. Persons with lived experience were individuals with current or previous experiences of mild-to-moderate anxiety, depression, and/or stress who had participated in a municipal NBHI. They contributed experiential knowledge during stakeholder dialogue meetings but were not involved in the more extensive iterative co-production activities due to considerations regarding vulnerability and participant burden.

Three stakeholder dialogue meetings were conducted to explore how research evidence could be translated into practice and to identify areas where experiential knowledge was needed to address gaps in the literature, with a total of 28 participants across the meetings [16]. The thematic analysis of data from these meetings generated seven overarching themes: target group needs, the added value of nature, outcome measurement, competency requirements, characteristics of the natural environment, organisational structures, and broader societal potential [22].

A two-round Delphi study with research experts was conducted to refine and prioritise key elements of NBHIs across four domains: target group, professional competencies, mechanisms of change, and outcomes. A detailed report of the Delphi study is provided elsewhere [16]; key findings are summarised here. The highest level of agreement concerned core mechanisms, including interaction with nature, social community, and physical activity, as well as key outcomes representing mental well-being, quality of life, and symptom reduction. Consensus among respondents also highlighted the importance of multidisciplinary professional competencies, while greater variation was observed in relation to group composition and delivery formats. Qualitative responses throughout the Delphi process further emphasised the need to balance group heterogeneity, adapt competencies to context, and align outcome selection with intervention aims.

### 2.4. Target Group

Based on inputs from stakeholder dialogue meetings and the Delphi process, ‘mild to moderate anxiety, depression, and/or stress’ is used as a practice-informed descriptor rather than a formal diagnostic category. The designation is grounded in professional judgement within municipal service contexts and does not rely on standardised diagnostic criteria or severity thresholds. The target group was defined functionally as individuals whose anxiety-, depression-, or stress-related difficulties constituted the primary cause of loss of everyday functioning, thereby distinguishing intervention-relevant conditions from transient or normative life stress.

### 2.5. Outcomes from Stage 1

Based on inputs from the literature review, stakeholder dialogue meetings, and the Delphi process, a set of core outcome domains was defined to reflect both intervention-relevant change and feasibility within practice contexts. These included mental well-being, quality of life, connection to nature, and symptoms of depression, anxiety, and stress. A core outcome battery was identified to provide a nuanced and holistic assessment of participant development and intervention impact, comprising the WHO-5 for mental well-being, the Satisfaction with Life Scale for overall life satisfaction, the Environmental Identity Scale for connection to nature, and the DASS-21 for symptoms of depression, anxiety, and stress [23,24,25,26]. Together, these measures capture complementary dimensions of change beyond symptom reduction, while remaining feasible for use in routine service settings. The selection further reflects the current limitation that validated instruments tailored to the target population and context remain scarce.

As described in a recent systematic review, a wide range of measurement instruments has been used in NBHIs, reflecting that the field is still emerging and characterised by a lack of consensus regarding measurable outcomes [8]. Mental well-being and quality of life were included to capture broader, salutogenic changes beyond symptom reduction, while symptoms of depression, anxiety, and stress ensured clinical relevance and comparability with existing mental health interventions. Within the well-being domain, the WHO-5 was included as a well-established and widely used instrument for assessing mental well-being. Satisfaction with Life was included as a more global measure of subjective well-being, as it is less influenced by specific life domains that are not expected to change as a result of the intervention. This allows for a more direct assessment of whether the intervention relates to participants’ overall evaluations of their lives and may, in this context, function more appropriately than multidimensional quality-of-life scales, which are more strongly influenced by life domains not expected to change as a result of the intervention. Connection to nature was included as an intervention specific outcome reflecting hypothesised mechanisms of change in NBHIs. The measurement tool Environmental Identity Scale was selected to assess whether and how the intervention was associated with changes in participants’ relationship with the natural environment. This choice was partly based on the scale’s broad inclusion of different types of natural environments, such as urban nature and rural natural environments, whereas other commonly used scales tend to focus more strongly on wild nature. Finally, the DASS-21 scale was selected, as it assesses stress, anxiety, and depression simultaneously, which was relevant given that several participants experienced combinations of mental health challenges, such as stress and depression. Using the DASS-21 allowed for a single, comprehensive measure to be applied consistently across participants.

Collectively, these activities generated an integrated evidence base comprising research findings, identified knowledge gaps, practice-informed insights, and expert consensus (Figure 1). Overall, the output from Stage 1 functioned to establish shared knowledge that was used to inform the co-production workshops (Stage 2) and the initial programme theory.

### 2.6. Stage 2: Co-Production Workshop

Stage 2 focused on translating the scientific foundation into a practice-oriented, implementable programme theory and logic model. A structured co-production workshop was conducted to develop and prototype a logic model capable of contextual adaptation when needed. The workshop enabled participants to articulate and critically examine how core mechanisms, intervention activities, and contextual conditions were assumed to relate within a coherent programme theory.

**Figure 2:**
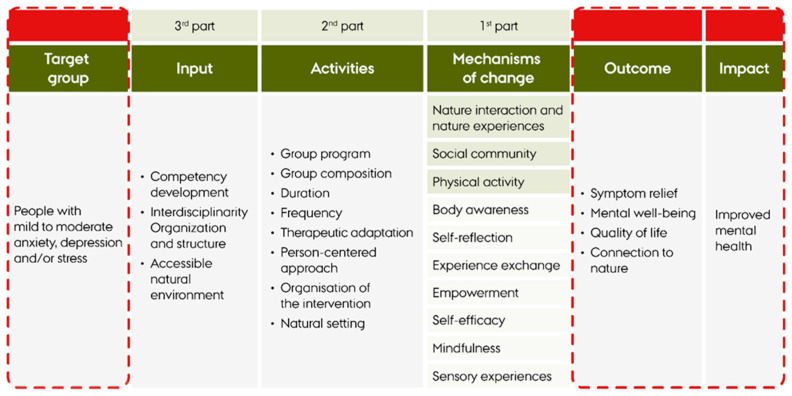
Logic model structure illustrating Stage 1-defined boundary elements and Stage 2 components for co-production. The red-lined predefined elements functioned as analytical boundary conditions for the workshop, ensuring continuity while delimiting the scope of co-production.

While the overall structure of the logic model, including the specification of the target group and intended outcomes, had been defined based on Stage 1 outputs, the intermediate components remained open for discussion and refinement (see Figure 2).

Workshop participants were invited to critically examine and refine the preliminary assumptions derived from Stage 1, particularly regarding:

- Core mechanisms of change supported by existing evidence (e.g., nature interaction, social connectedness, physical activity);
- The specific activities required to activate these mechanisms;
- The contextual preconditions necessary for successful implementation.

Through facilitated group exercises, participants explored how these elements were expected to align and interact.

### 2.7. Workshop Participants

The workshop brought together intervention practice partners with direct experience in delivering nature-based activities across mental health, social care, community, and psychiatric settings, alongside project researchers. This configuration was intended to integrate research-informed assumptions with experiential and practice-based knowledge grounded in real-world implementation contexts. A total of 11 participants took part in the co-production workshop (Table 1). Two practitioners from each intervention site were invited (n = 6), alongside five project researchers who participated on equal terms in the collaborative process. This composition was purposefully chosen, prioritising practitioners directly involved in delivery and ensuring that feasibility and contextual applicability remained central to the co-production process.

### 2.8. Co-Production Workshop Design and Procedure

The workshop followed a structured, component-wise progression, enabling systematic examination and refinement of how research-informed assumptions aligned with practice-based knowledge. The workshop began with a brief presentation of Stage 1: Scientific foundation, highlighting the evidence and theory behind the preliminary logic model (Figure 2). This clarified which elements were fixed and which could be refined.

Two members of the research team facilitated the workshop, accompanied by an intern who documented the proceedings, using the visual modelling software tool MIRO, which supported the joint identification and positioning of mechanisms, activities, and inputs within a shared logic model framework. Contributions from each group exercise were integrated in real time, allowing participants to collectively position, revise, and examine relationships between components. This facilitated immediate clarification of assumptions, identification of gaps or overlaps, and shared validation of the emerging programme theory. The emphasis was not on designing a fixed intervention model, but on articulating a theoretically coherent and context-adaptive programme theory illustrated through a logic model that could subsequently be adapted to local contexts.

The core of the workshop comprised three sequential group sessions focusing on: (1) mechanisms of change, (2) activities and core elements, and (3) inputs and structural preconditions (Figure 2). Each session followed the same format: presentation of draft material, small-group deliberation (3–4 participants), and plenary consolidation within the shared logic model, facilitated with MIRO. Participants were encouraged to make implicit assumptions explicit, assess contextual feasibility, and refine conceptual distinctions.

The workshop concluded with a plenary review of the collectively assembled logic model, examining internal coherence, plausibility of causal pathways, and alignment across components. Throughout the process, provisional elements were iteratively refined, enabling the negotiation of shared understandings while anchoring the developing programme theory in both empirical evidence and implementation experience.

### 2.9. Stage 3: Prototyping and Context Adaptation

Stage 3 focused on (1) the prototyping of a programme theory including the logic model and (2) contextual adaptation of the Nature Impact Mental Health Intervention, including procedures and intervention materials. This involved the analytical consolidation and practical operationalisation of the collaboratively developed programme theory, as illustrated in a logic model.

To ground the programme theory in existing practice contexts, observation of current practice was conducted with each of the three intervention practice partners. Two researchers visited each site and spent one full day together with the local delivery team observing current practice and discussing contextual conditions relevant for implementation, including characteristics of the natural environment, competency needs among staff, and practical considerations for a forthcoming feasibility test. In addition, continuous supervision was provided.

Based on these visits, draft manuals and associated materials were synthesised into a consolidated intervention catalogue structured around the specified mechanisms, intervention activities, and contextual conditions. This process enabled systematic examination of internal coherence, practical feasibility, and alignment between theoretical assumptions and implementation requirements across settings.

For further preparation ahead of piloting [27] (not included in the current paper) and to follow the development of the intervention, local intervention adaptation through a one-day workshop was conducted with the delivery teams from all three intervention practice partners to further adapt and refine the intervention components for each site. In addition, professional competency development was performed through a 2-day workshop held with the delivery teams to support preparation for implementation, laying the groundwork for a subsequent feasibility study.

## 3. Results

The co-production process resulted in a set of clearly defined and interrelated mechanisms of change and intervention activities, that formed the programme theory of the Nature Impact Mental Health Intervention. These were iteratively specified and refined through co-production workshops (Stage 2) and further developed during prototyping (Stage 3), where relationships between components were clarified, assumptions made explicit, and conceptual coherence strengthened.

The resulting programme theory is presented in three parts: first, the identified mechanisms of change; second, the key intervention activities; and, finally, the consolidated logic model, which provides the foundation for subsequent context-adapted feasibility testing of the intervention (see Appendix A).

### 3.1. Mechanisms of Change

In line with the Medical Research Council (MRC) Framework 2021 [17], the programme theory defines mechanisms of change as the processes through which the intervention produces its effects, reflecting how participants engage with and respond to core activities. Within this study, particular emphasis was placed on how these mechanisms are activated in practice and how they interact.

Nature interaction and nature experience were specified as inherently linked to sensory experience, emphasising how engagement with the natural environment occurs through embodied and sensory processes. Physical activity was reconceptualised from a potentially clinical or performance-oriented understanding towards a broader notion of movement, where bodily engagement in nature, rather than intensity or measurable outputs, was considered central. Social community was emphasised as a core structural element, highlighting the importance of group-based formats and the active facilitation of social processes within intervention activities.

The additional mechanisms were discussed in relation to their positioning, meaning, and interrelations within the programme theory and logic model. In contrast to the three core mechanisms, which were understood as directly translatable into intervention activities, these were conceptualised as emerging through engagement in such activities. For example, physical activity in nature was described as fostering body awareness, while social community facilitated experience sharing. Mechanisms such as mindful presence, body awareness, experience sharing, and self-reflection were thus understood to support and reinforce the core mechanisms. Self-efficacy, empowerment, and transfer to everyday life were, in contrast, conceptualised as underlying processes embedded in professional facilitation, aimed at supporting participants’ agency and engagement beyond the intervention.

The following descriptions present the core mechanisms of change as part of the resulting programme theory and detail how they are understood to operate within the intervention. This is followed by a synthesis of the additional mechanisms and their role in supporting and shaping the overall logic model.

#### 3.1.1. Nature Interaction and Sensory Experience

Interaction with nature and engagement of the senses constitute a central mechanism of change. Through direct, embodied interaction with natural environments, participants may develop a deeper sense of relationship with nature, understood as an embodied and emotional sense of connection to the natural environment [28,29,30]. Relatedness to nature has been associated with enhanced mental well-being, including lower levels of stress, anxiety, and depressive symptoms, and may function as a psychological resource supporting resilience and emotional regulation.

Active engagement with the natural environment shifts attention away from ruminative and self-focused thought patterns towards embodied sensory experience, supporting a “return to the body” and fostering present-moment awareness. This shift in attention is particularly relevant for individuals experiencing stress-related or affective disorders, where persistent rumination and self-focused attention often dominate experience.

Through direct and sustained interaction with natural surroundings, participants are exposed to a rich and varied multisensory input, including tactile contact with natural elements, movement across uneven terrain, and continuous exposure to natural sounds and visual stimuli.

It is suggested that natural environments can, depending on their quality, provide a distinctive context for developing positive bodily experiences and body awareness through embodied interaction with nature [31]. These multisensory experiences may support the development of interoceptive awareness, understood as the capacity to perceive and interpret internal bodily signals such as breathing, heart rate, and muscle tension. Interoceptive awareness has been associated with improved emotional regulation, as it enables individuals to recognise early signs of stress and respond more adaptively [32].

Both planned activities and spontaneous encounters with nature are used therapeutically, enabling participants to explore, reflect, and derive insights, while facilitators guide the process without overshadowing the direct interaction with the natural environment.

#### 3.1.2. Physical Activity

Physical activity in natural environments is a key mechanism linking bodily engagement with mental and social well-being [33,34]. Movement is embedded into the environment, using natural features to promote strength, balance, flexibility, and coordination. Activities are designed not as direct transfers of indoor exercises, but as interactions with the environment, such as navigating trails, balancing on logs, or engaging with natural obstacles.

Physical activity supports mental health by reducing negative effects, fatigue, and stress, while enhancing energy, attention, and mood [35]. Integrating movement into outdoor activities also fosters engagement, enjoyment, and motivation, allowing participants to experience both challenge and mastery in a safe and supportive context. Structured partner or group activities further link physical movement with social interaction and reflection, creating a holistic pathway through which NBHIs promote sustained mental health benefits.

#### 3.1.3. Social Community

Social community is a core mechanism of change, fostered by shared experiences in the natural environments [34]. Nature provides a neutral, informal context where group cohesion can emerge organically. Participants may experience support, trust, and mutual understanding, whether through direct interaction, collaboration on tasks, shared reflection, or simply through co-presence and parallel engagement in activities.

Facilitators play a crucial role in balancing opportunities for individual participation and social engagement, ensuring that participants who prefer observation or limited interaction can still benefit. The environment acts as a ‘common third,’ supporting collaboration and collective problem-solving, such as joint navigation, building a fire, or shared exploration. By allowing participants to contribute actively and take ownership of tasks, facilitators enhance self-efficacy, empowerment, and transferable skills, which are essential for sustaining gains beyond the intervention and applying learned strategies in daily life.

#### 3.1.4. Additional Mechanisms at Play

Additional mechanisms were identified as supporting and reinforcing the core mechanisms of change, adding depth to the overall programme theory. These included present-moment awareness, experience exchange, body awareness, and self-reflection.

Present-moment awareness was understood as an overarching state of awareness characterised by attention directed towards experiences in the here and now [13,14]. It enabled participants to disengage, at least temporarily, from distressing or ruminative thought patterns. This state was closely intertwined with body awareness, understood as awareness of bodily movement, sensory input, and emotional reactions [13,14]. It was described as strengthening participants’ ability to attend to and interpret bodily signals, supporting emotional regulation, and contributing to a closer integration of body and mind.

Experience exchange was closely linked to social community, providing opportunities for participants to reflect on and share personal experiences [36]. This process was understood to support mutual recognition and the development of coping strategies. Self-reflection enabled participants to process experiences, identify personal resources, and develop new understandings of their situation [37]. Interaction with the natural environment was described as facilitating reflection by evoking associations, memories, and new perspectives.

Together, these additional mechanisms were understood to enhance participants’ capacity for meaning-making, emotional regulation, and engagement with everyday life, thereby reinforcing the overall programme theory.

### 3.2. Activities

In line with the MRC Framework 2021, activities were conceptualised as the key components through which the intervention’s mechanisms of change are activated [17]. While specific settings and exercises may vary across contexts, three core activities were identified as essential to support the intended outcome of the intervention: therapeutic adjustments, choice of natural environments, and organisation of the intervention.

During the co-production workshop, preliminary assumptions regarding intervention activities (Figure 2) were refined and organised into broader analytical categories, reflecting key aspects of implementation and the role of health professionals in operationalising the programme theory. Therapeutic adjustment emerged as a central component of the intervention, highlighting the importance of the delivery team and the facilitation of activities in achieving impact and realising the therapeutic potential of the natural environment. Foundational frameworks such as the biopsychosocial and person-centred approaches were emphasised as essential for translating the intervention into outdoor settings [38]. The intervention was further characterised as a multidisciplinary effort, requiring a therapeutic approach capable of accommodating diverse professional perspectives.

Observations of current practice with partners provided insights into existing delivery formats and site-specific conditions, including team composition and recruitment pathways. These informed the local adaptation of the intervention and the design of professional competency development for delivery teams. This development focused on strengthening the integration of body and mind, identifying activities aligned with the core mechanisms, and preparing delivery teams for implementation through supervision and discussion of key aspects such as recruitment, initial consultations, and team dynamics.

The selection of natural environments was discussed with particular attention to accessibility and the interpretation of key characteristics such as variation and biodiversity. Participants emphasised that environments should be chosen based on locally available conditions, prioritising qualities that promote safety, calm, and sensory engagement. These qualities were not understood as fixed or idealised criteria, but rather as context-dependent and relative to what is available in each setting. Accordingly, environments were selected based on their suitability and diversity within specific local contexts. This understanding was further refined through observations of current practice with partners, where discussions were grounded in the concrete environments available at each location. These visits enabled a more nuanced and practice-oriented understanding of how requirements related to natural settings could be operationalised.

The organisation of the intervention programme was shaped by practical considerations related to implementation across settings. Programme duration was considered within a range of 6 to 12 weeks, taking into account factors such as participants’ connection to the labour market, capacity for change, and opportunities for progression. Group size and format were identified as key components. Groups were considered to require 8–12 participants to sustain group dynamics, while accounting for expected dropout and absence. Session length was understood as context-dependent and closely linked to participant needs. Factors such as energy levels, need for disengagement, and tolerance for social interaction were highlighted as influencing how sessions should be organised across settings.

The three activity categories are presented in turn, detailing how each contributes to the operationalisation of the programme theory in practice, including its logic model.

#### 3.2.1. Therapeutic Adjustments

Therapeutic adjustments constitute a central element of the intervention. It was understood as a continuous and responsive practice in which professional judgement is shaped in relation to how situations unfold [39]. Rather than being guided by standardised procedures, professional practice was characterised by flexibility and adjustments to participants’ needs, resources, and vulnerabilities. In this way, healthcare professionals continuously adjusted activities and interactions in response to the specific individual, group and context.

A person-centred approach guides the selection and progression of activities across physical, cognitive, and social dimensions, accommodating fluctuations in mental state and functional capacity [38]. The intervention is further grounded in a biopsychosocial perspective, integrating physiological regulation, psychological processes, and social interaction within the group [40]. Therapeutic adjustment also incorporates a focus on self-efficacy and empowerment through manageable challenges, mastery experiences, and active participation [41,42]. The facilitation by health professionals was understood to support the transfer of experiences and strategies to everyday life, thereby promoting sustained engagement beyond the intervention [43]. Psychoeducation is integrated throughout the process, using dialogue and experiential learning to support understanding of mental health and the role of nature as a therapeutic resource [44].

#### 3.2.2. Choice of Natural Environment

The choice of the natural environment constitutes a central component of the intervention, where the environment is understood as an active therapeutic element rather than a passive setting. Natural environments are selected to support mental well-being while affording a range of therapeutic activities.

Drawing on established perspectives such as Attention Restoration Theory and the Biophilia hypothesis, key characteristics guiding the choice of natural environment include variation in landscape, opportunities for both shelter and openness, and sensory impressions such as sounds, textures, and light conditions [13,45,46]. Environments offering perceived biodiversity and opportunities for exploration are considered particularly valuable for supporting engagement and reflection. In this context, stable and recognisable base points, such as sheltered gathering areas or landmarks, provide containment and predictability, enabling participants to engage safely with more challenging areas of the environment.

The interaction between activities and the natural environment is central to therapeutic impact. Both structured activities and spontaneous encounters with nature are used to facilitate reflection, meaning making, and insight. In this context, nature is described as a co-therapeutic element, providing a setting in which participants engage bodily, emotionally, and cognitively, while facilitators support the integration of these experiences within the therapeutic process [47].

#### 3.2.3. Organisation of the Intervention

**Figure 3:**
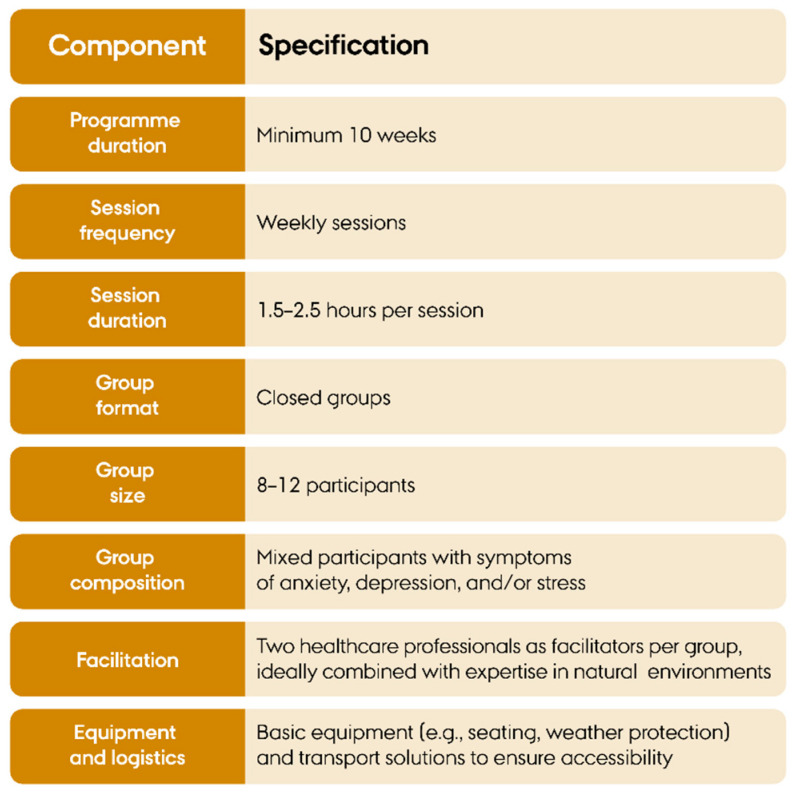
Organisational components of the Nature Impact Mental Health Intervention.

The organisational frame of the intervention provides the structural conditions necessary for consistent implementation across contexts. Key parameters, such as duration, group format, and facilitation, are standardised (Figure 3) based on considerations of feasibility, participant needs, and the activation of core mechanisms.

The organisation of the intervention is formed to support building trust, continuity, and engagement over time. Closed group formats and stable participation structures facilitate group cohesion and psychological safety, while the involvement of two health professional facilitators ensures both safety in outdoor settings and the possibility for individualised support within group-based activities. The intervention framework reflects a balance between standardisation and flexibility, allowing adjustments to participant needs and local conditions while maintaining consistency in the delivery of the intervention.

**Figure 4:**
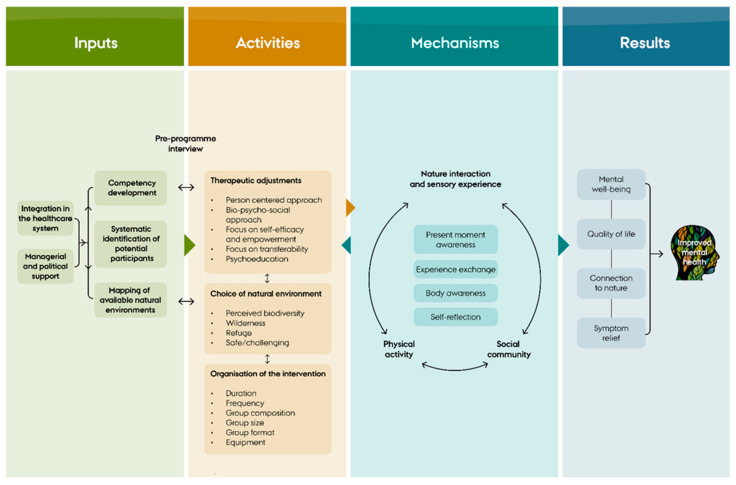
Co-produced prototype of the Nature Impact Mental Health Intervention logic model.

Together, the identified mechanisms and activity domains are integrated and visually synthesised in the co-produced logic model (Figure 4). The model represents the consolidated outcome of the development process, illustrating how specific intervention activities are expected to activate certain mechanisms of change and, in turn, contribute to short- and longer-term mental health outcomes. It also makes explicit the underlying assumptions and hypothesised causal pathways linking intervention components to outcomes, thereby enhancing the transparency and theoretical coherence of the programme theory.

## 4. Discussion

This study provides a co-produced programme theory including a logic model for an NBHI targeting people experiencing mild to moderate anxiety, depression, and/or stress. Despite a growing body of research demonstrating the positive effects of nature on mental health, the field remains characterised by limited theoretical integration and fragmented approaches to intervention design [48]. This study therefore provides a novel contribution to the field by offering a theoretically grounded and practice-informed framework to guide subsequent feasibility testing, and ultimately the development, implementation, and evaluation of NBHIs in real-world healthcare contexts.

Furthermore, the logic model serves as a practical framework to guide implementation and evaluation in subsequent context-adapted feasibility testing across the three practice partners. In this regard, it provides a shared reference point for assessing intervention fidelity, identifying context-specific adaptations, and examining how and for whom the intervention produces its intended effects.

### 4.1. Strengths and Limitations of the Nature Impact Mental Health Intervention

A key strength of the Nature Impact Mental Health Intervention lies in its intentional design as a flexible yet structured framework, where core components are preserved while allowing for systematic local adaptation. Rather than treating adaptation as a pragmatic necessity, the Nature Impact Mental Health Intervention explicitly integrates context-sensitivity into its design, enabling delivery teams to tailor activities, settings, and facilitation to local conditions while maintaining the integrity of the underlying programme theory. This approach aligns with established perspectives on programme theory, which emphasise the importance of clearly articulating underlying mechanisms to support adaptation across contexts while still achieving intended outcomes [49]. By providing explicit descriptions of both the programme theory and the accompanying logic model, this study offers a transparent framework for understanding how mechanisms of change are expected to operate in practice, thereby supporting both implementation and future evaluation.

At the same time, this approach engages with ongoing debates regarding the limitations of logic models in representing complex, context-sensitive interventions [50,51]. It has been argued that traditional logic models may struggle to capture how interventions adapt in real-world settings and that more flexible and dynamic approaches are required [50]. In this context, the present study demonstrates how a logic model can be used not as a fixed representation, but as a structured yet adaptable tool that supports both consensus-building and context-sensitive implementation. This is particularly relevant in the field of NBHIs, which are inherently complex and multi-component, where full standardisation is neither feasible nor desirable [52]. As a result, there is a need for approaches that accommodate variation while maintaining coherence. The present study contributes to this by offering a set of core principles and structures that enable local adaptation without compromising the theoretical and therapeutic integrity of the intervention. In doing so, it supports implementation across diverse contexts and enhances the potential transferability of NBHIs within real-world healthcare settings.

However, several limitations should be acknowledged. A central limitation is that the programme theory and its underlying mechanisms remain theoretically derived and have not yet been empirically validated. While the study articulates—based on a three-stage co-production process—how mechanisms such as nature interaction and sensory experiences, physical activity, and social community are expected to influence mental health outcomes, further research is needed to examine how these mechanisms operate in practice, including how additional mechanisms are internally related and linked to the core mechanisms [53].

The Nature Impact Mental Health Intervention was developed within a Danish context, and although designed to allow for local adaptation, its transferability to other settings may require further contextual tailoring. In addition, the intervention is intended for implementation across municipal healthcare centres and outpatient hospital contexts, which may introduce heterogeneity and complicate comparisons across settings [17]. Accordingly, while the intervention is designed to be transferable, its effectiveness and implementation are likely to depend on context, highlighting the need for careful and systematic adaptation, for example, guided by frameworks such as the ADAPT (Adapting interventions to new contexts) guidance [54].

In addition, while the ambition to develop a generic, transdisciplinary model supports flexibility and broader applicability, it may also be perceived as insufficiently specific for individual disciplines. Nonetheless, this approach deliberately seeks to challenge siloed practices by encouraging professional autonomy and responsibility within a collaborative transdisciplinary framework.

Finally, the co-production approach, involving both users and practitioners, strengthened the intervention’s alignment with user needs and clinical expertise, which may enhance engagement, adherence, and long-term sustainability. The inclusion of multiple stakeholders and insights from cross-national perspectives further contributed to the relevance and robustness of the intervention. However, we acknowledge recent discussions regarding the use of the term ‘stakeholders’, with critiques highlighting its potentially problematic connotations and proposing ‘interest-holders’ as a more neutral alternative that better reflects individuals’ or groups’ legitimate interests in health research and policy [55]. Nevertheless, we retain the term ‘stakeholders’ to ensure consistency with the MRC framework and its established use in health research [17]. The inclusion of multiple stakeholders and cross-national perspectives further enhances the relevance and robustness of the intervention.

An additional consideration concerns how nature is conceptualised within NBHIs. The intervention was not developed from a perspective that views nature solely as a therapeutic resource for human benefit. Rather, connection to nature was included as both a mechanism and intervention-specific outcome, reflecting a relational understanding of the human–nature relationship. Although environmental stewardship was not explicitly explored, stronger connections to nature may have implications beyond individual health outcomes.

In addition to the strengths and limitations related to the intervention itself, several methodological considerations should be acknowledged. A key strength lies in the systematic involvement of practitioners and practice partners throughout the three-stage co-production process, ensuring that the intervention is locally grounded, and practice relevant. The iterative co-production approach further enabled early identification of potential barriers and continuous refinement of intervention components, thereby increasing the likelihood of feasibility, acceptability and engagement in real-world settings.

However, limitations should also be noted. The evidence base underpinning the proposed mechanisms of change in NBHIs remains limited, which constrains the extent to which the programme theory and its logic model can be anchored in well-established causal pathways. In addition, while co-production enhances contextual relevance, it may introduce variability in intervention components across sites, potentially complicating future evaluations of effectiveness. Finally, as with co-produced interventions more broadly, balancing scientific rigour with practical relevance remains an ongoing challenge.

### 4.2. Implications

The development of a co-produced, theory-informed NBHI offers several implications for research and practice. For researchers, the articulated programme theory including the general logic model provides a structured foundation for subsequent empirical testing, particularly through feasibility studies that can assess acceptability, implementation processes, and preliminary outcomes. Such studies are especially warranted given the limited evidence on the mechanisms underpinning NBHIs and the contextual adaptations embedded in the intervention.

For practitioners, the co-production approach illustrates how interventions can be designed to align with local contexts and delivery capacities, potentially enhancing feasibility and uptake in routine practice. More broadly, this work underscores the potential of NBHIs as a complementary approach to mental health support, while highlighting the need for stepwise evaluation. A logical next step is to conduct a feasibility study to examine implementation in practice and to inform the design of future effectiveness trials. In addition, a prototype intervention catalogue has been developed alongside the framework. This will be further refined based on findings from the forthcoming feasibility study and is intended to support structured adaptation of intervention content to different contexts and target groups. In the longer term, the catalogue may serve as a practical guide for practitioners implementing similar NBHIs in other settings.

For decision makers, this study offers practice-relevant knowledge to inform the development and implementation of NBHIs within existing health and social care systems. The integration of empirical insights with a clear programme theory supports evidence informed decisions related to feasibility, implementation, and scaling. Thereby it becomes clear how nature-based approaches can be embedded in practice to promote mental health and well-being.

Finally, the intervention was deliberately developed and delivered across multiple types of natural environments, including a therapeutic garden, a forest setting, and an urban nature area. This multicontextual approach highlights how NBHIs can be flexibly adapted to different landscape types and degrees of urbanisation, while maintaining core intervention principles. By engaging with both designed and managed natural environments, the programme illustrates how diverse green spaces may support mental health interventions through various sensory qualities, affordances for movement, and opportunities for social engagement. These findings underscore the relevance of considering environmental context as an active component of NBHI design and evaluation, aligning with interdisciplinary research across health, environmental psychology, and landscape-based studies. The present study does not allow firm conclusions regarding whether depression, anxiety, or stress are differentially sensitive to NBHIs, and this remains an interesting area for future research, particularly in relation to mechanisms of change and outcome specificity.

## 5. Conclusions

This study demonstrated a transparent and systematic process for co-producing a programme theory and logic model for NBHIs. Through active stakeholder involvement and an explicit linking of theory, practice, and context, the study developed a model that not only clarified the expected mechanisms of change but also highlighted the implementation conditions critical for success within real-world healthcare systems.

The resulting model provides a theoretically grounded and implementation-sensitive foundation for subsequent feasibility testing. Its transparency enabled the identification of key assumptions, potential barriers, and contextual dependencies that should be further explored in future research. This strengthened both the internal validity of the intervention and its transferability across settings.

In addition, the study offered methodological guidance on how NBHIs could be integrated into existing healthcare systems through co-production, iterative development, and clear operationalisation of the relationships between inputs, activities, outputs, and outcomes. This approach may serve as a practical framework for researchers and decision-makers working to design, adapt, and implement complex NBHIs.

## Figures and Tables

**Table 1:** Co-production workshop participants.

ID	Title	Profession	Experience with NBHIs
ID 1	Practitioner	Nurse	>10 years
ID 2	Practitioner	Social worker	5–10 years
ID 3	Practitioner	Physiotherapist	<5 years
ID 4	Practitioner	Psychotherapist	<5 years
ID 5	Practitioner	Physiotherapist	<5 years
ID 6	Practitioner	Nurse	<5 years
ID 7	Researcher	Medical Doctor	<5 years
ID 8	Researcher	Psychologist	<5 years
ID 9	Researcher	Physiotherapist	>10 years
ID 10	Researcher	Anthropologist	5–10 years
ID 11	Researcher	Physical science	<5 years

**Figure A1:**
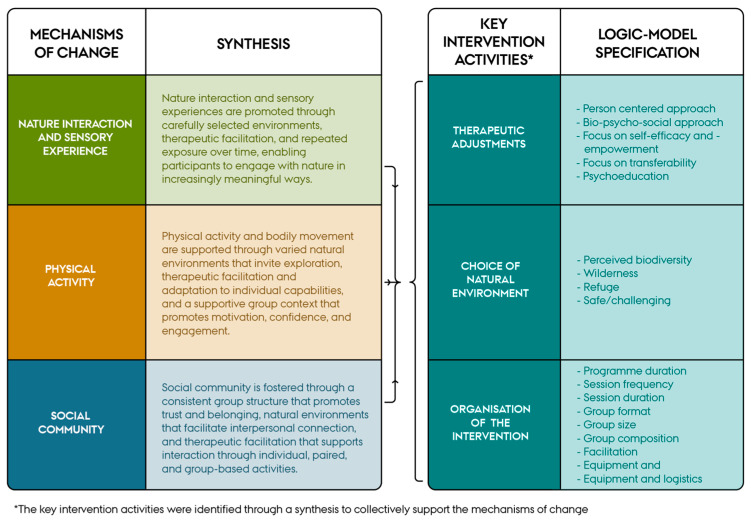
The development process, illustrating how the identified mechanisms of change informed the key intervention activities and intervention specifications.

## Data Availability

The data presented in this study may be available on request from the corresponding author due to the privacy of the respondents.

## Abbreviations

The following abbreviations are used in this manuscript:

NBHI	Nature-based health intervention
MRC	Medical Research Council
